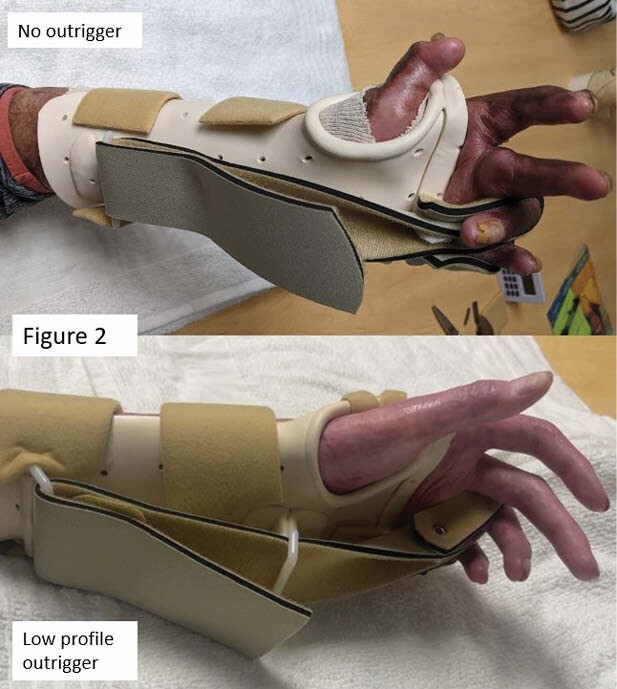# 564 Simplified Dynamic Splints for Common Scar Contractures of the Hand

**DOI:** 10.1093/jbcr/irad045.160

**Published:** 2023-05-15

**Authors:** Gregory Andre, Brooke Dean, Scott Vocke, Joshua Rodriguez

**Affiliations:** Johns Hopkins Bayview Medical Center, Lutherville- Timonium, Maryland; Johns Hopkins Bayview Medical Center, Catonsville, Maryland; Johns Hopkins Bayview Medical Center, Baltimore, Maryland; Johns Hopkins Bayview Medical Center, Timonium, Maryland; Johns Hopkins Bayview Medical Center, Lutherville- Timonium, Maryland; Johns Hopkins Bayview Medical Center, Catonsville, Maryland; Johns Hopkins Bayview Medical Center, Baltimore, Maryland; Johns Hopkins Bayview Medical Center, Timonium, Maryland; Johns Hopkins Bayview Medical Center, Lutherville- Timonium, Maryland; Johns Hopkins Bayview Medical Center, Catonsville, Maryland; Johns Hopkins Bayview Medical Center, Baltimore, Maryland; Johns Hopkins Bayview Medical Center, Timonium, Maryland; Johns Hopkins Bayview Medical Center, Lutherville- Timonium, Maryland; Johns Hopkins Bayview Medical Center, Catonsville, Maryland; Johns Hopkins Bayview Medical Center, Baltimore, Maryland; Johns Hopkins Bayview Medical Center, Timonium, Maryland

## Abstract

**Introduction:**

Common problems associated with immature burn scarring of the hands include proximal interphalangeal (PIP)/distal interphalangeal (DIP) flexion contracture, first webspace contracture, and metacarpal phalangeal (MCP) hyperextension that require diligent splinting intervention in therapy and at home to improve patient outcomes. Dynamic splinting can be used to provide low-load long-duration stretch; however, they can be difficult for patients to don independently at home and are often time consuming for therapists to fabricate. Alternative methods of using neoprene strapping were utilized to provide the dynamic pull to simplify the supplies required and improve ease of use for the patient.

**Methods:**

Using the “I splint” for elbow flexion contractures as a model, we fabricated a smaller version for use in finger PIP and DIP flexion contractures using thermoplastic material and neoprene strapping (Figure 1a). A novel splint for thumb abduction/extension in treatment of first web space scar contracture (Figure 1b). Based on a traditional MCP flexion splint, we utilized neoprene strapping instead of springs or rubber bands and a plastic D ring instead of metal outrigger (Figure 2).

Splint wear started at 1x/ day for 5 minutes and progressed to 3-5x/day for 5-15 minutes as tolerated.

**Results:**

Digit PIP or DIP extender (figure 1a.) after 1 week of daily wear improved PIP extension from 45 degrees (of flexion) to 25 degrees and DIP extension from 50 degrees to 30 degrees.

Thumb Post Splint (figure 1b.) after 2 weeks of daily wear improved radial abduction from 45 degrees to 58 degrees.

Dynamic MCP flexion splint (figure 2) after 1 month of daily wear improved left ring finger active MCP flexion from 0-20 degrees to 0-40 degrees. Left small finger active MCP flexion improved from (-50) - 0 degrees to (-40) - 5 degrees (negative parenthetical value denotes hyperextension).

**Conclusions:**

Dynamic splints utilizing neoprene strapping contributed to improved range of motion for all patients included in this case series. Therapists noted that splint adjustments were infrequent and minimal throughout time of use. Patients reported ease of use and compliance with splint wear schedule outside of therapy sessions. The amount of time spent fabricating compared to traditional methods was not tracked and would be an interesting point to consider in the future.

**Applicability of Research to Practice:**

We hope that this presentation may give other practitioners new tools to address common burn scar contractures of the hand and simplify both the fabrication process and materials required for dynamic splinting.